# Preparation and Electrochemical Performance of Bio-Oil-Derived Hydrochar as a Supercapacitor Electrode Material

**DOI:** 10.3390/ijerph20021355

**Published:** 2023-01-11

**Authors:** Juntao Wei, Jiawei Sun, Deliang Xu, Lei Shi, Miao Wang, Bin Li, Xudong Song, Shu Zhang, Hong Zhang

**Affiliations:** 1 Joint International Research Laboratory of Biomass Energy and Materials, Co-Innovation Center of Efficient Processing and Utilization of Forest Resources, College of Materials Science and Engineering, Nanjing Forestry University, Nanjing 210037, China; 2 School of Energy and Power Engineering, Jiangsu University, Zhenjiang 212013, China; 3 State Key Laboratory of High-Efficiency Utilization of Coal and Green Chemical Engineering, Ningxia University, Yinchuan 750021, China

**Keywords:** hydrothermal, nitrogen doping, KOH activation, bio-oil, electrochemical performance

## Abstract

The rapid consumption of fossil energy and the urgent demand for sustainable development have significantly promoted worldwide efforts to explore new technology for energy conversion and storage. Carbon-based supercapacitors have received increasing attention. The use of biomass and waste as a carbon precursor is environmentally friendly and economical. In this study, hydrothermal pretreatment was used to synthetize coke from bio-oil, which can create a honeycomb-like structure that is advantageous for electrolyte transport. Furthermore, hydrothermal pretreatment, which is low in temperature, can create a low graphitization degree which can make heteroatom introduction and activation easier. Then, urea and KOH were used for doping and activation, which can improve conductivity and capacitance. Compared with no heteroatom and activation hydrothermal char (HC) (58.3 F/g at 1 A/g), the prepared carbon material nitrogen doping activated hydrothermal carbon (NAHC_1_) had a good electrochemical performance of 225.4 F/g at 1 A/g. The specific capacitance of the prepared NAHC_1_ was improved by 3.8 times compared with that of HC.

## 1. Introduction

The rapid consumption of fossil energy and the urgent demand for sustainable development have greatly promoted worldwide efforts to explore new technology for energy conversion and storage [1]. Supercapacitors, which are energy storage devices, have many advantages compared with traditional storage devices, such as high power density, long lifecycle, fast charge and discharge performance, etc. Therefore, supercapacitors have attracted increasing interest among many researchers. The storage types of supercapacitors include (1) electric double-layer capacitance; (2) and pseudo capacitance [2]. The mechanism of electric double-layer capacitance is that the ion is stored based on physical electrostatic adsorption without any chemical reaction during charging and discharging [3]. The mechanism of pseudo capacitance is that charge storage occurs via a fast reversible redox reaction on or near the surface [4]. For electric double-layer capacitors (EDLCs), the carbon materials are widely applied [5]. Biomass and waste [6,7,8], including onions, houttuynia, lotus stalks, bio-oil, etc., are significant sources or precursors to carbon materials due to their high carbon content and low cost. For example, some researchers [9] use yeast as a carbon precursor to prepare electrode material, which has good electrochemistry performance that has 316 F g^−1^ at 1 A g^−1^.

However, the performance of carbon derived from biomass is significantly low for its complex structure and chemical composition. Many researchers seek methods to enhance the electrochemical performance of the materials. Widely used means of modification involve activation and doping.

The activation treatment of materials is the most effective way to enhance performance by raising materials’ specific surface area. Methods of activation treatment are usually divided into two kinds, namely chemical activation and physical activation [10]. Chemical activation is the most convenient and effective method, which generally uses potassium hydroxide (KOH) [11], sodium hydroxide (NaOH), salt, etc. Chemical activation can produce a high specific surface area and porous structure. Wang et al. [12] employed KOH as an activator to activate carbon from Metaplexis japonica. The sample has an ultrahigh surface area of about 2210 m^2^ g^−1^ and high specific capacitance of 287 F g^−1^ at 1.0 A g^−1^. Wang et al. [13] used a mixture of NaCl and ZnCl_2_ activators to create a surface area of about 484 m^2^ g^−1^ and the sample had a specific capacitance of 293 F g^−1^. Due to the great electrode performance of the heteroatom doping carbon, some researchers have applied doping to enhance electrochemical performance. Nitrogen (N) [14], sulfur (S) [15], and boron (B) [16] are generally used as the doping element. There are two methods of doping heteroatoms: one is self-doping and the other is chemical doping. Self-doping [17] involves the heteroatoms of electrode materials being introduced by raw materials. Chemical doping [18] involves the heteroatoms of electrode materials being introduced by using other sources. Owing to the size of nitrogen compared with that of carbon, nitrogen doping is the most common method in the heteroatoms doping of electrode materials [19]. Because nitrogen heteroatoms in porous carbon can improve surface wettability and electronic conductivity and induce pseudo capacitance, they thus promote the capacitance of supercapacitors [20]. Many researchers have employed nitrogen doping to improve the electrochemical performance of carbon materials. For example, Wu et al. [21] used lilac and lotus seedpods as carbon precursors and N_2_ nonthermal plasma generated by the dielectric barrier discharge (DBD) to finally synthesize nitrogen doping carbon materials which had a great electrochemical performance of 342.5 F/g at 0.5 A/g.

Bio-oil is the important product of biomass pyrolysis, and contains a large number of aromatic hydrocarbons and polycyclic carbon compounds [22], which can be easily polymerized and aromatized to generate carbons with heat treatment. Thus, bio-oil is a desirable carbon precursor to synthesize electrode materials. Compared with biomass, bio-oil shows obvious advantages such as low ash content [23] and liquid phase. Accordingly, electrode material derived from bio-oil have better stability and easy modification. Additionally, bio-oil shows high oxygen content. Oxygen content is advantageous to modify the surface electron properties of carbon materials and improve the electrode wettability to increase ion-accessible surface areas [24], which can enhance solid–liquid interface reaction and thus improve the electrochemical performance. Therefore, it can be concluded that bio-oil-derived carbon is a promising choice for supercapacitor electrode material. Prior to actual application, the production of bio-oil-derived carbon is the key. Some researchers [25,26] have successfully used hydrothermal carbonization (HTC) to upgrade bio-oil into a solid fuel, and it was found that HTC was conducive to enhancing carbon content in hydrochar and removing the unstable component in bio-oil through dehydration and decarboxylation reaction [25]. Furthermore, compared with the conventional carbonization method (i.e., pyrolysis or high-temperature carbonization), HTC shows incomparable advantages such as low energy consumption due to its mild reaction conditions, high solid product yield [26], and high oxygen content functional groups on solid surface. Therefore, bio-oil-derived hydrochar might be an attractive supercapacitor electrode material. However, the related study on preparation and electrochemical performance of bio-oil-derived hydrochar as a supercapacitor electrode material was almost a gap.

In this work, a nitrogen doping, porous bio-oil-derived carbon electrode materials were efficiently synthesized. Firstly, bio-oil was treated by hydrothermal pretreatment. Subsequently, nitrogen was introduced into hydrochar using urea. The nitrogen doping carbons were activated by KOH. The morphology of the prepared samples was charactered by SEM. The surface area and pore structure of the samples were analyzed by BET. The graphitization degree of the samples was characterized by XRD and Raman. The doping nitrogen type of the sample was characterized by XPS. The electrochemical performance was characterized by electrochemical workstation at 6 M KOH electrolyte. The final synthesized sample had a good electrochemical performance of 225.4 F/g at 1 A/g.

## 2. Materials and Methods

### 2.1. Hydrothermal Pretreatment of Bio-Oil

The feedstock of this study was fast pyrolysis bio-oil (provided by Biomass Technology Group, Enschede, The Netherlands. An amount of 60 g of fast pyrolysis bio-oil (FPBO) was directly poured into a stainless steel kettle. The reactant was subsequently heated to 250 °C at 300 r/min under argon (Ar) atmosphere and then kept at 250 °C for 8 h. The product was cooled to room temperature and then washed by ethanol 5–6 times, which was denoted as HC. The mass yield of HC was about 28.3%.

### 2.2. Synthesis of N-Doped Carbon from HC

The N-doped carbon was synthesized by carbonizing a mixture of urea and HC. In detail, 2 g HC and 2 g urea were mixed in an agate mortar. The mixture was placed on a quartz boat. Afterwards, the quartz boat was placed into a tubular furnace. The reaction temperature was 700 °C for 1 h with a heating rate of 5 °C/min. Argon (Ar) was served as the carrier gas in the tubular furnace. The product was cooled to room temperature. The product was washed by deionized water 5–6 times. The product was subsequently was dried at 105 °C for 24 h. The product was named NHC_1_. Similarly, the as-prepared N-doped carbon was named NHC_2_, NHC_3_.

### 2.3. Synthesis of N-Doped Porous Carbon from NHC

N-doped porous carbon NHCs were activated by KOH. In detail, 3 g of KOH was dissolved in deionized water and then 3 g of NHC was added into the solution. The mixed solution was stirred for 2 h at room temperature. After being stirred, the mixed solution was dried at 105 °C for 24 h. the mixed solid powder was placed into the tubular furnace. The reaction temperature was 700 °C for 1 h with a heating rate of 5 °C/min. Argon (Ar) was served as the carrier gas in the tubular furnace. The product was cooled to room temperature. The product was first washed with 0.5 M HCl and then washed with deionized water for 5–6 times. The product was subsequently dried at 105 °C for 24 h. The product was named NAHC_1_, NAHC_2_, NAHC_3_.

### 2.4. Characterization

Nitrogen adsorption–desorption isotherm measurements were used to analyze the pore structure of samples (BSD-PM4). The surface area and pore size distribution were determined by using the Brunauer–Emmett–Teller theory and the nonlocal density functional theory method, respectively. The morphologies, microstructures, and crystallinity of the samples were analyzed by Quanta 200 scanning electron microscopy (SEM, FEI, Hillsboro, Ultima, IV, USA), X-ray diffraction (XRD, Rigaku, Tokyo, Japan), AXIS UltraDLD X-ray photoelectron spectroscopy (XPS, Shimadzu, Kyoto, Japan), and DXR532 Raman scattering spectroscopy (Raman, ThemorFisher, Waltham, MA, USA )

### 2.5. Supercapacitor Electrode Preparation and Assembly 

The prepared samples were mixed with a conductive carbon black and PTFE emulsion (mass ratio = 8:1:1) in alcohol. The prepared slurry was applied evenly on circular nickel foam to form an electrode after drying [27].

### 2.6. Electrochemical Measurements

The electrochemical performance of the samples electrode was tested by a three-electrode system. In a typical three-electrode system, the as-prepared electrode, Pt foil and Hg/HgO were used as the working electrode, counter electrode, and reference electrode, respectively. A total of 6 M KOH was used as electrolyte. An electrochemical working station was used for the cyclic voltammetry (CV), galvanostatic charge–discharge (GCD), and impedance measurements.

The specific capacitance of a single electrode was calculated according to the galvanostatic charge–discharge curve using Equation (1) for the three-electrode system [27]:

(1) $$C_{m}=\frac{I\Delta t}{m\Delta V}$$

where C_m_ is the specific capacitance (F/g), *I* (A) is the discharge current, Δt (s) is the discharge time, *m* (g) is the total mass of active materials in both electrodes, and ΔV (V) is the potential change within the discharge time.

## 3. Results and Discussions

### 3.1. Morphology and Pore Structure

**Figure 1:**
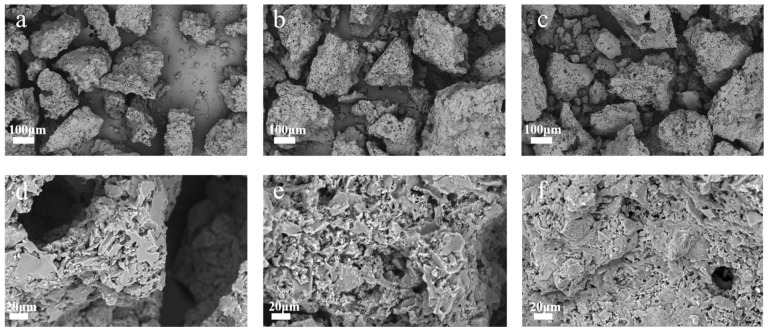
SEM image of (**a**) NHC_1_, (**b**) NHC_2_, (**c**) NHC_3_, (**d**) NAHC_1_, (**e**) NAHC_2_, and (**f**) NAHC_3_.

The SEM images of the samples were shown in Figure 1. All images of the samples presented a sheet structure. Due to high pressure and polarity of water in bio-oil, the bio-oil precursor was transformed to honeycomb-like coke during the hydrothermal process. Honeycomb-like carbon materials are good for the transmission of electrolytes [28]. It was observed that the sheet structure of NAHC_1_ was destroyed as a result of KOH activation. The activation effect of KOH can improve a specific surface area of the sample, while also making the sheet collapse and furthermore, causing a block in electrolyte transmission. It can be seen in Figure 1a–c that the surface of NHCs were slightly destroyed. It can be inferred that the high temperature and urea pressure vapor destroyed the surface of the sample during the urea vaporization process. After KOH activation, the destruction obviously increased, as seen in Figure 1d,f. With the ratio of urea increasing, the destruction was more obvious, which indicates that the doping of nitrogen would decrease the graphitization degree; thus, the effect of KOH activation was better.

**Figure 2:**
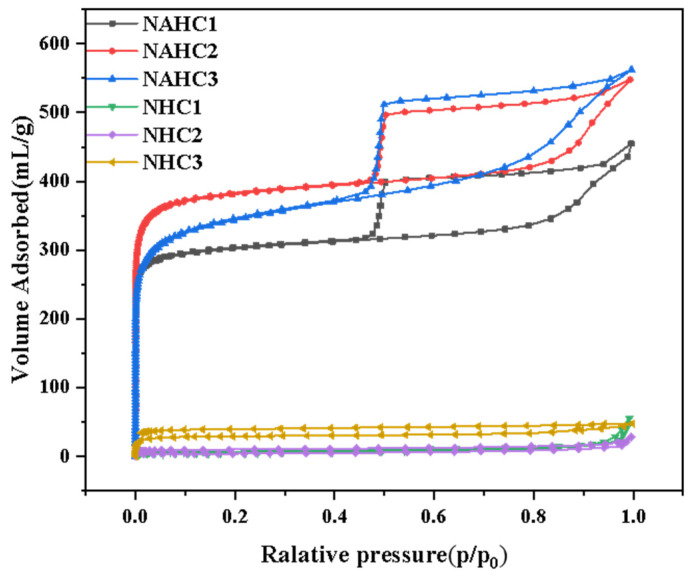
N_2_ adsorption–desorption isotherms of various samples.

The N_2_ adsorption–desorption measurement was conducted to analyze the porous structure. The nitrogen adsorption–desorption isotherms of NHC_1_ and NAHC_1_ were shown in Figure 2. The nitrogen adsorption–desorption isotherms of NHCs show a type-IV [29] isothermal with low S_bet_ of 15–16 m^2^/g, which would seriously affect electrochemical performance. Since the samples were activated by KOH, the pore structure of samples was obviously improved. The nitrogen adsorption–desorption isotherms of all NAHCs were shown a combination of type I, II, and IV [30], with an obvious hysteresis loop at a relative pressure range of 0.4 and 1, which indicates the existence of a hierarchical pore structure. It was confirmed that micropore and mesopore, especially mesopores, can improve electrochemical performance [31]. The detailed pore structure parameter was shown in Table 1. NAHC_1_ had the largest S_meso_ (229.2837 m^2^/g). Although NAHC_1_ did not have the largest S_Bet_ or nitrogen content, the electrochemical performance of NAHC_1_ was the best, as discussed in the following section.

### 3.2. Carbon Structure and Elemental Composition 

**Figure 3:**
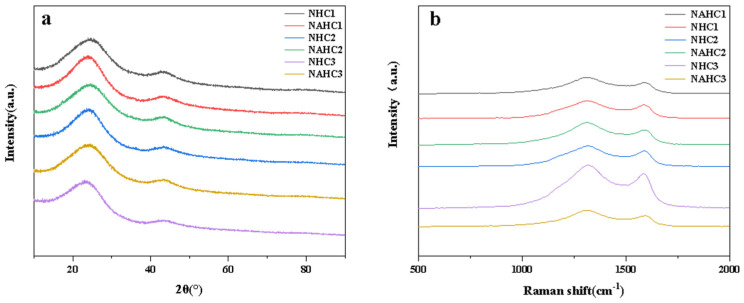
(**a**) XRD pattern of NHCs and NAHCs; (**b**) Raman spectra of NHCs and NAHCs.

Figure 3a shows the XRD pattern of the NHCs and NAHCs. it shows two wide peaks located at ~24° and ~43° in Figure 3a, which assigns the graphitic crystallite plane (002) and crystallite plane (100) [32]. Compared with NHCs, the peak (002) of NAHCs was broader, which revealed that the graphitization degree of NAHCs became weaker than that of NHCs. Thus, it can be inferred that the activation treatment reduces the graphitization degree of NHCs, which can be proven by Raman spectroscopy.

The Raman spectroscopy was applied to analyze the graphitization of the samples. Two signals, 1350 cm^−1^ and 1580 cm^−1^, can be observed in Figure 3b, which represents the D band and G band [33], respectively. These characteristic D and G bands of the carbon materials represent disordered structures and sp^2^-bonded carbon atoms [34] (E_2g_ mode of 2D graphite), respectively. The intensity ratio of the D/G bands (I_D_/I_G_) was used to characterize the degree of defects [35]. The higher I_D_/I_G_ indicated more defects in the samples. More defects mean a better electrochemical performance. The exhibited ratio I_D_/I_G_ of NHC_1_, NAHC_1_, NHC_2_, NAHC_2_, NHC_3_, and NAHC_3_ was 1.286, 1.472, 1.320, 1.510, 1.279, and 1.568. The NHCs samples exhibited a lower ratio of I_D_/I_G_ than the NAHCs, which implied that the effect of the KOH activation treatment would reduce the degree of the graphitization. With the increasing ratio of nitrogen, the ratio of I_D_/I_G_ of NAHCs also increased. The highest degree of defects was detected in the NAHC_3_ sample. The results suggest that: (1) a KOH activation treatment and nitrogen doping treatment would increase the ratio of I_D_/I_G_ of the material; (2) and with the increasing content of nitrogen, the ratio of I_D_/I_G_ also increased.

**Figure 4:**
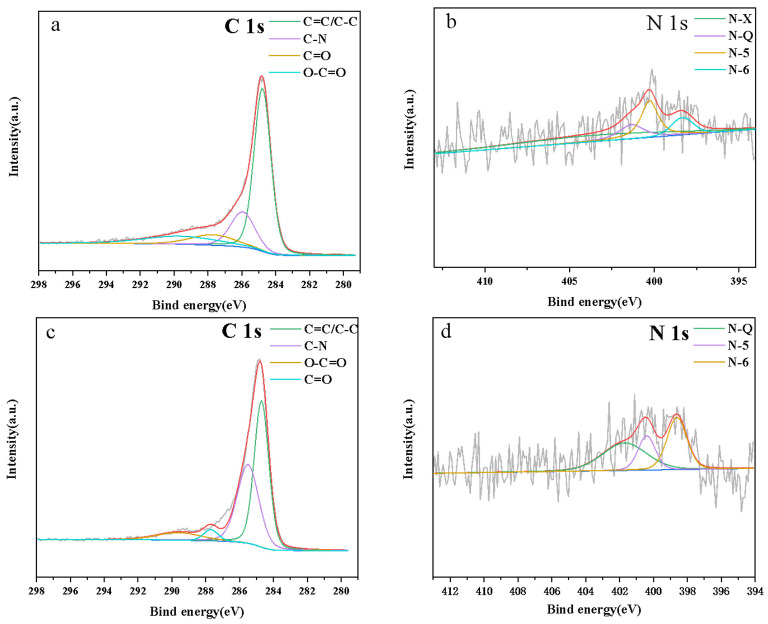
(**a**) C 1s core level of XPS Spectrum of NHC_1_; (**b**) N 1s core level of XPS Spectrum of NHC_1_; (**c**) C 1s core level of XPS Spectrum of NAHC_1_; (**d**) N 1s core level of XPS Spectrum of NAHC_1_.

X-ray photoelectron spectroscopy was applied to investigate the modality of nitrogen doping. Table 2 shows all the samples’ elemental compositions. Due to the effect of KOH activation, the nitrogen contents of all samples were decreased. It was inferred that KOH activation had essential damage to the nitrogen-containing structure. Figure 4a,c shows the high-resolution XPS C 1s of the NHC_1_ and NAHC_1_. The C 1s spectrum can be divided into four fitted peaks, 284.5, 285.7, 287.7, and 289.6 eV [36], which represent C-C/C=C, C-N, C=O, and O-C=O, respectively. The N 1s spectrum form in Figure 4b,d can be divided into four and three fitted peaks corresponding to NHC_1_ and NAHC_1_, respectively. The N 1s spectrum of NHC_1_ contains four peaks, 398.1, 400.1, 401.8, and 403.5 eV [34], which are assigned to the pyridinic-N(N-6), pyrrolic-N(N-5), quaternary-N(N-Q), and pyridinic-N-oxide(N-X), respectively. However, the N 1s spectrum of NAHC_1_ only contains three peaks, which are N-6, N-5, and N-Q. Compared with the N 1s of NHC, it can be seen that the intensity of NAHC’s N-X was disappeared and the intensity of N-5, N-6, and N-Q was enhanced. Due to the existence of π-conjugated skeletons, N-5, N-6, and N-Q were stable in the activation treatment of NHC [37]. Moreover, N-X would be destroyed in the activation treatment. the previous research proved that N-5 and N-6 were expected to provide active sites to enhance electrochemically performance [38]. Furthermore, the previous work proved that N-Q can enhance the wettability of the electrode and N-5 and N-6 can improve electron mobility [39].

### 3.3. Electrochemical Performance

**Figure 5:**
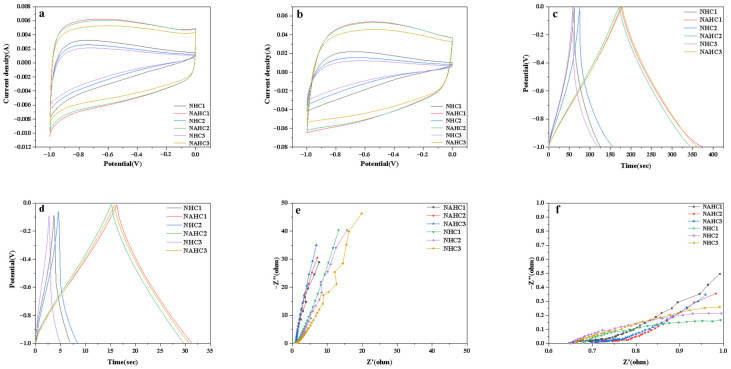
CV curves of tested samples at (**a**) 10 mV/s and (**b**) 100 mV/s; GCD curves of tested sample at (**c**) 1 A/g and (**d**) 10 A/g; (**e**) Nyquist plots of tested samples; (**f**) the partial enlarged view of the Nyquist plots.

To characterize the electrochemical performance of NHCs and NAHCs, CV and GCD curves were measured in an aqueous electrolyte solution of 6 M KOH using a three-electrode system [30]. CV curves were tested 5 cycles before it was collected, and each sample was tested 3 times. As shown in Figure 5a, the CV curves at 10 mV/s of NAHCs show quasi-rectangular shapes, which revealed a quick electrochemical response and dominant electric double-layer capacitance behavior [40]. A weak and broad peak can be observed, which may be derived from the reversible redox reaction due to the existence of nitrogen [41]. The integral area of the CV curve indicates the electrode’s ion adsorption capacity. The integral area of NHCs’ CV curve shows a low size, which indicates a weak electrode’s ion adsorption. It can be inferred that a low specific surface area would be adverse to an electrode’s ion adsorption. Nevertheless, the integral area of the NAHCs’ CV curve was large and it was increased with the decrease in doping ratio. By the above BET measurement, it was found that the high specific surface area can improve an electrode’s ion adsorption. The CV curves at 100 mV/s show a similar trend in Figure 5b; although the peak was more obvious, the curves kept a quasi-rectangular shape at 100 mV/s.

Capacitive performance was evaluated by a GCD measurement. The GCD curves of all samples displayed a nearly straight line and are symmetrical in Figure 5c, which can imply that the samples represent a dominant electric double-layer capacitance (EDLCs) behavior and a good charge–discharge process. The time of the charge–discharge process reflected the capacitance of samples by the above formula, the specific capacitance can be calculated. Among NHCs, NHC_2_ had the highest specific capacitance, which was 82.8 F/g at 1 A/g. NHC_1_ and NHC_3_ had close specific capacitances, which were 65.2 F/g and 63.2 F/g at 1 A/g, respectively. Through KOH activation, specific capacitances had a huge enhancement. NAHC_1_ had the highest specific capacitance, which was 210.5 F/g at 1 A/g. The specific capacitance of NAHC_1_ was 3.2 times higher than NHC_1_, which illustrated that enhancement of a specific surface area would improve the electrochemical performance. The GCD curves of all samples at 10 A/g were shown in Figure 5d. NAHC_1_ maintained good electrochemical performance, which was 151 F/g. The specific capacitance of other samples, especially NHCs, seriously decreased.

An EIS measurement was employed to study the resistance and capacitance of samples. At the low frequency region in Figure 5e, it can be seen that the more inclined a line is close to the theoretical vertical line, the better the capacitance performance of the samples are. In Figure 5e, the lines of NAHCs are closer to the theoretical vertical line [40], especially NAHC_3_; therefore, NAHCs have better capacitance. The lines of NHCs were not good even in not straight lines, which may be due to the poor pore structure of NHCs. At the high frequency region shown in Figure 5f, NAHCs and NHCs all have a low internal resistance (Rs) (0.648–0.704 Ohm) value and charge transfer resistance (Rct) due to the doping of the nitrogen which can improve hydrophilicity and the transmission performance of electrolytes in the materials.

**Figure 6:**
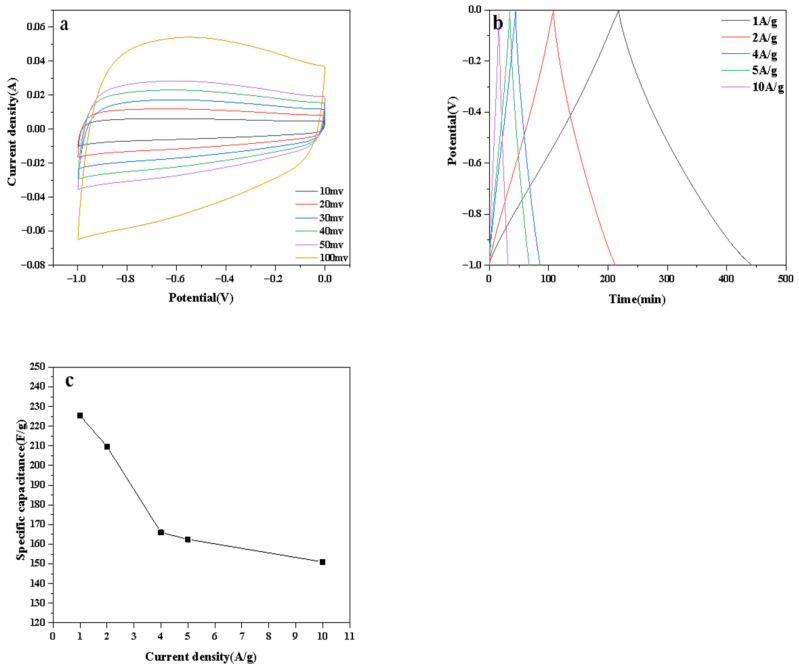
(**a**) CV curves, (**b**) GCD curves, and (**c**) the capacitance of NAHC_1_.

To further explore the performance of NAHC_1_, CV and GCD were employed in Figure 6. In Figure 6a, all CV curves from 10 mV/s to 100 mV/s were quasi-rectangular in shapes, which indicated that NAHC_1_had EDLC behavior from 10 mV/s to 100 mV/s. With the sweep rate increasing, the peak of NAHC_1_ was more obvious due to the enhancement of pseudo capacitance. In Figure 6b, the time of charge and discharge process were decreased with the increase in the current density. By calculating capacitance, the capacitance of NAHC_1_ was decreased with the increase in current density in Figure 6c. At a low current density, decreasing the capacitance was not obvious. However, it badly decreased at a high current density. Capacitance at 1 A/g was 225.4 F/g, while capacitance at 10 A/g was 151 F/g. The capacitance of NAHC_1_ at 10 A/g remained at 67%. Table 3 shows the performance comparison of NAHC_1_ with other biomass carbon materials.

## 4. Conclusions

In this study, different kinds of carbon material from bio-oil were fabricated through hydrothermal pretreatment, nitrogen doping, and KOH activation. The NHCs had a honeycomb-like structure, which promoted the transmission of electrolytes. However, the NHCs had a poor pore structure and a specific surface area of just 14.3789 m^2^/g; thus, the specific surface of NAHCs was enhanced greatly after KOH activation, which had a specific surface area of 1163.3115 m^2^/g. The NAHCs had a lower graphitization degree and I_D_/I_G_ than the NHCs. The nitrogen content was improved by nitrogen doping. NAHC_1_ had a nitrogen content of 3.47%. The nitrogen doping types of NAHC_1_ were mainly pyridinic-N(N-6), pyrrolic-N(N-5), and quaternary-N(N-Q), which contribute to the enhancement of the electrochemical performance. Compared with other NAHCs, NAHC_1_ had a higher nitrogen content. Thus, NAHC_1_ had the best electrochemical performance of 225.4 F/g at 1 A/g. Compared with other NAHCs, NAHC_1_ had nitrogen content. Then NAHC_2_ had an electrochemical performance of 183.3 F/g and NAHC_3_ had an electrochemical performance of 175.3 F/g at 1 A/g. As a result of poor structure, NHCs had a very low electrochemical performance of 63.2–82.8 F/g at 1 A/g. In conclusion, carbon material electrochemical performance factors are varied. In this study, the optimal synthesis sample was NAHC_1_. 

## Figures and Tables

**Table 1:** Porosity parameters of tested samples.

Sample	Pore Structure Parameter				
	S_Bet_ (m^2^/g)	S_mcro_ (m^2^/g)	S_meso_ (m^2^/g)	V_mcro_ (m^3^/g)	V_meso_ (m^3^/g)
NHC_1_	15.13	3.84	11.29	0.04	0.03
NHC_2_	14.38	2.99	11.39	0.01	0.06
NHC_3_	16.53	0	16.53	0	0.08
NAHC_1_	1175.46	946.18	229.28	0.44	0.43
NAHC_2_	1420.17	1319.06	101.11	0.55	0.29
NAHC_3_	1163.31	1079.82	83.50	0.44	0.24

**Table 2:** Elemental contents of tested samples measured by XPS.

Sample	Elemental Composition		
	C (At.%)	N (At.%)	O (At.%)
NHC_1_	81.77	3.55	14.69
NHC_2_	82.19	3.63	14.18
NHC_3_	82.06	3.78	14.16
NAHC_1_	91.68	2.93	5.39
NAHC_2_	90.80	2.84	6.36
NAHC_3_	90.01	3.30	6.69

**Table 3:** Various precursors based on activated carbon as supercapacitor electrodes.

Carbon Precursor	C_g_ (F/g)	Electrolyte	Ref.
Coal tar pitch	143	6 M KOH	[42]
Poly(vinyl alcohol)	210	6 M KOH	[43]
Dairy manure	202	6 M KOH	[44]
Setaria viridis	199	6 M KOH	[45]
Lumpy bracket	223	6 M KOH	[46]
Glebionis coronaria	205	6 M KOH	[47]
Bio-oil	225.4	6 M KOH	This work

## Data Availability

The data presented in this study are available on request from the corresponding author.
